# The experience of beauty derived from sorrow

**DOI:** 10.1002/hbm.23657

**Published:** 2017-05-23

**Authors:** Tomohiro Ishizu, Semir Zeki

**Affiliations:** ^1^ Wellcome Laboratory of Neurobiology, Department of Cell and Developmental Biology University College London London United Kingdom; ^2^ Japan Society for the Promotion of Science Tokyo Japan

**Keywords:** aesthetic experience, emotion, functional magnetic resonance imaging

## Abstract

We studied the neural mechanisms that are engaged during the experience of beauty derived from sorrow and from joy, two experiences that share a common denominator (beauty) but are linked to opposite emotional valences. Twenty subjects viewed and rerated, in a functional magnetic resonance imaging scanner, 120 images which each had classified into the following four categories: beautiful and sad; beautiful and joyful; neutral; ugly. The medial orbito‐frontal cortex (mOFC) was active during the experience of both types of beauty. Otherwise, the two experiences engaged different parts of the brain: joyful beauty engaged areas linked to positive emotions while sorrowful beauty engaged areas linked to negative experiences. Separate regions of the cerebellum were engaged during experience of the two conditions. A functional connectivity analysis indicated that the activity within the mOFC was modulated by the supplementary motor area/middle cingulate cortex, known to be engaged during empathetic experiences provoked by other peoples' sadness. *Hum Brain Mapp 38:4185–4200, 2017*. © **2017 Wiley Periodicals, Inc.**

*You came to me to learn the Pleasure of Life and the Pleasure of Art. Perhaps I was chosen to teach you something much more wonderful, the meaning of Sorrow, and its beauty*

Oscar Wilde (1896),

(in a letter to ‘Bosie’, Lord Alfred Douglas)

## INTRODUCTION

Over the past few years, we and others have addressed the question of what neural mechanisms are engaged during aesthetic experiences, and especially during the experience of beauty. In addressing the question, we were inspired by the question posed by the English art critic, Clive Bell. In his book *Art* [1914], Bell asked whether there is anything common to all objects that are experienced as beautiful or that arouse the aesthetic emotion. Translating this into neural terms we, likewise, sought to understand whether there is a common brain system in which activity correlates with the experience of beauty. Surprising though it may seem, although the experience of beauty derived from different sources entails activity in different areas of the brain, depending upon the source, there is one common area, located in the medial orbito‐frontal cortex (mOFC) of the emotional brain, in which activity correlates parametrically with the experience of beauty, whether derived from sensory sources such as music or visual art [e.g., Ishizu and Zeki, 2011; Kawabata and Zeki, 2004], from moral sources [Tsukiura and Cabeza, 2011; Wang et al., 2015] or from highly cognitive sources such as mathematics [Zeki et al., 2014]. In the work reported here, we were inspired by the quote from Oscar Wilde given above, to explore the neural activity that correlates with the experience of beauty derived from sorrow. The quote implies that sorrowful beauty belongs in a separate, or separable, category, which can be defined as the experience of a positive (aesthetic) emotion with a negative component, that of sadness. To study the neural correlate of such an experience, we had to incorporate another distinct category—that of beauty aroused from joy, which can be categorised as a positive emotion with a positive component—for comparison.

Important though the distinction between sorrowful and joyful beauty is, it is one that is not often made, or not made emphatically enough. This is surprising because the distinction is easily recognized and experienced by most, even if both categories arouse the aesthetic emotion. In sculpture, for example, Michelangelo's great *Pietà* in Rome is suffused with pathos, tenderness and sadness, whereas the *Three Graces* of Canova are joyful and playful. In music, the waltzes of Johann Strauss are light‐hearted and engaging while the adagio from Beethoven's Ninth Symphony is permeated with contemplative sorrow. The list is endless and includes works in literature, poetry, dance, and theatre. Photography, especially, has provided many examples that can be easily classified emotionally by any viewer as sorrowful or joyful, with beauty as their common denominator; good examples are Dorothea Lange's iconic images of the Great Depression, and especially *The Migrant Mother*, on the one hand and Bill Brandt's *East End Girl Dancing* on the other. Aside from beauty, these two separate categories share another common denominator, empathy, which makes it possible for humans to become aware of the feelings of others and indeed experience those same feelings—whether of joy or sorrow—to varying degrees.

It seemed to us that this distinction provides fertile ground for a neurobiological enquiry into the brain mechanisms that are engaged when two contrasting affective states, a negative one (sorrow) and a positive one (joy) both result in the experience of beauty. This enquiry parallels, in a sense, our previous enquiry into the distinction, in neural terms, between the sublime and the beautiful, two categories that have been discussed much more extensively in philosophies of aesthetics. The common description of the sublime as containing a negative affect (‘pleasure from displeasure’ or ‘beauty mingled with horror’) is reflected in a pattern of brain activity that is different from that which is engaged during the experience of the beautiful [Ishizu and Zeki, 2014]. This made it interesting to enquire whether we can also detect differences in the pattern of brain activity during aesthetic experiences derived from two opposite states. Our general hypothesis was that there would be profound differences with the two experiences but that, given the pre‐eminence of activity in the mOFC during the experience of the beautiful, the latter would be active in both states, even in spite of the evident differences between the two. Moreover, since experiencing beauty derived from positively or negatively valenced emotion inevitably requires mentalizing others' emotional states or interpreting their intentions, empathy is another common denominator to the experience of beauty in sorrowful and joyful sources. We, thus, expected to find activity in brain regions which past studies have implicated in empathetic experiences.

## MATERIALS AND METHODS

### Participants

Twenty one healthy right‐handed volunteers (11 females; 10 males; mean age, 28.6 years) from different cultures and ethnic backgrounds (2 Taiwanese, 4 Indian, 6 Japanese, 2 Middle Eastern, and 7 West and North Europeans) participated but data from one volunteer was excluded because of excessive noise during scanning, leaving us with 20 volunteers; all had normal or corrected‐to‐normal vision, and none had a history of neurological or psychiatric disorder. Written informed consent was obtained from all and the study was approved by the Ethics Committee of University College London, and conformed to the Code of Ethics of the World Medical Association (Declaration of Helsinki). All data were anonymized.

### Preliminary Psychophysical Testing and Postscanning Ratings

Although pictures depicting war scenes, weapons, violence or strong political, and religious attributes may often be deemed sorrowful or beautiful, we excluded them from this study because it is known that viewing scenes depicting violence, or which remind viewers of violence, can induce immediate brain responses, such as activity within frontal and limbic system, even when viewed passively (e.g., Kelly et al., 2007]; we wanted to exclude such automatic brain responses which are unrelated to the current task. We, therefore, used instead pictures of events like funerals, abandoned children and buildings, and sad faces, as well as landscapes and daily scenes, in both monochrome and in color. The 800 pictures that we used were drawn from photographic magazines and books, including *The Family of Man* (in which the photographs referred to in the Introduction can be found), *The Modern Century*, and *The Great LIFE Photographers*.

During a first visit to the laboratory, between 3 and 7 days prior to scanning, each subject was instructed about the experiment and, in a psychophysical test, rated the stimuli according to their aesthetic and emotional valence through two questionnaires. In the aesthetic evaluation, participants classified a picture into five groups according to the intensity of the aesthetic experience aroused in them, using a Likert scale extending from 5 (‘very beautiful’) to 1 (very ugly), with 3 being ‘neutral’; in the emotional evaluation, 5 corresponded to ‘very joyful’, 3 to ‘neutral’, and 1 to ‘very sorrowful’. Thus, we obtained, for each subject, an emotional and an aesthetic rating for each of the 800 stimuli. Participants gave the aesthetic and the emotional ratings in counterbalanced order. It should be noted that they were instructed to give emotional ratings according to the feelings that they experienced when viewing the images, not to those of what people in the images might feel.

Each stimulus remained on the computer screen until participants responded to the second evaluation, after which an inter‐trial interval of 1 s followed; they were then asked to press a button as soon as possible after they had made their evaluation and were also asked to indicate their familiarity with each picture (‘have you seen this picture before?’) and familiar pictures were excluded.

**Figure 1:**
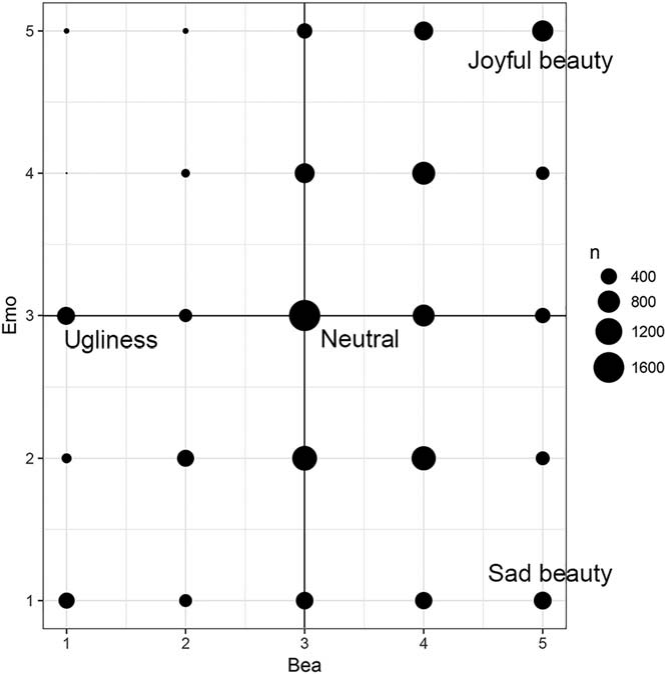
Preliminary behavioral data summed over 20 subjects. Frequency distribution of beauty rating (*x* axis) versus emotion rating (*y* axis). Size of each circle is proportional to the number of trials for that rating.

From these 800 rated pictures, we selected, for each subject, 120 which fell into the four categories of ‘sorrowful beauty’, ‘joyful beauty’, ‘neutral’, and ‘ugly’. Pictures falling into the sorrowful beauty category were the ones that had been given a rating of 1 on the emotional score and 5 on the aesthetic score; pictures falling into the joyful beauty category had a rating of 5 on the emotional scale and 5 on the aesthetic scale while those rated as neutral had 3 on both scales. Stimuli rated as ugly had a score of 3 (neutral) for the emotional rating and 1 for the aesthetic score (see Fig. 1). Each of the four categories had 30 stimuli, making a total of 120 stimuli which each participant viewed in the scanner. We excluded five participants out of twenty‐six after the preliminary psychophysical tests showed that they did not have sufficient trials for each of the four categories. The detailed behavioral data obtained in the preliminary psychophysics are found in Supporting Information.

During the scanning session, participants were asked to rerate each picture aesthetically, after viewing it, but this time on a scale of 3 to 1 (beautiful, neutral, ugly). Immediately after scanning, they rerated the stimuli (which were presented in the same order as in the scanner) for their emotional valence, using a 3‐point Likert scale (3 as joyful, 2 as neutral, 1 as sorrowful).

### Paradigm and Procedure

Stimuli were generated using Cogent 2000 (http://www.vislab.ucl.ac.uk/cogent_2000.php) running in MATLAB (MathWorks, Natick, MA); they were back‐projected onto a screen using an LCD projector, through an angled mirror. The resolution of the screen was 1,400 × 1,050 pixels; the height of each stimulus was 19° while the width varied.

The session began with subjects viewing a flat black screen for 20 s to allow for T1 equilibration effects to subside (the corresponding first six brain volumes were discarded). After this 20 s blank period, an instruction about the aesthetic judgment appeared on the screen, to inform participants that a session had started. A fixation point then appeared at the centre of the screen for 1 s against a black background, after which visual stimuli were presented in a pseudorandom order for 6 s. We had 30 images in each of the four experimental conditions, making 120 images in total. We had six functional scanning sessions for each subject. Each functional session had 20 trials. To make a set of 20 images, we selected 5 images randomly out of 30 from each of the experimental conditions so that each condition had the same number of trials through a functional session. After this procedure, we randomized the sequence of stimulus presentation within a functional scanning. The stimulus presentation was followed by an interval with a jitter of 5–7 s, during which participants gave their aesthetic ratings.

Following each stimulus presentation, participants were asked to rate it on a 3‐point Likert scale, by pressing one of three buttons with their right index, middle or ring finger. The response period lasted 5–7 s and participants could make their rating at any time during that period; it ended with a blank period of 20 s, during which the scanner continued to acquire blood‐oxygen‐level dependent (BOLD) signals. The stimuli were presented in six sessions. Each session consisted of 20 stimuli with a 20 s resting period between the first and the last 10 trials during which participants were instructed not to close their eyes. Prior to the scanning, participants had a short practice session with a different set of visual stimuli to those used in the scanning session.

### Functional Magnetic Resonance Imaging Scanning

Scanning data were acquired in a 3‐T Siemens Magnetom Trio magnetic resonance imaging scanner (Siemens, Erlangen, Germany) fitted with a 32‐channel head‐coil. An echo‐planar imaging (EPI) sequence was applied for functional scans to obtain BOLD signals (echo time, 30 ms; repeat time, 3.36 s), using 48 slices to cover the whole brain. The voxel resolution was 3 × 3‐mm in‐plane resolution, with a 2 mm slice thickness and 1‐mm inter‐slice gap. Magnetic resonance imaging signal losses in the orbitofrontal cortex (OFC) and amygdala were reduced by applying a z shim gradient moment and slice tilt [Weiskopf et al., 2006]. T1‐weighted anatomical images were acquired at the end of the experimental sessions for each subject (176 slices; resolution, 1 × 1 × 1 mm; echo time, 2.48 ms; repeat time, 7.92 ms). Field maps were also acquired with the Siemens standard gradient‐echo field map sequence to correct for geometric distortion of EPI images [Hutton et al., 2002]. We also recorded the heart and respiration rates for each subject.

### Functional Magnetic Resonance Imaging Data Analysis

All data were analysed with SPM8 (Statistical Parametric Mapping, http://www.fil.ion.ucl.ac.uk/spm/software/spm8/). The EPI images for each subject were realigned and normalized into Montreal Neurological Institute (MNI) space, smoothed with a Gaussian smoothing kernel of 9 × 9 × 9 mm, and filtered with a high‐pass cut‐off (128 s) to remove drift terms. The stimulus for each subject was modelled as a set of regressors in a general linear model first‐level (within subject) analysis. The experiment was a block design, and boxcar functions were used to define the stimulations; these modelled the onsets and durations of the visual stimuli. Head movement parameters calculated from the realignment preprocessing step, physiological recordings, and response periods were included as regressors of no interest. Stimulus functions were convolved with a canonical haemodynamic response function. Contrast images were taken to random‐effects second‐level (between subject) analyses to produce statistical maps at the group level.

To carry out categorical contrast analyses according to the intensity of the aesthetic and emotional experience, ratings were coded as 1, 2, 3 for ‘ugly’, ‘neutral’, and ‘beautiful’, and 1, 2, and 3 for ‘sorrowful’, ‘neutral’, and ‘joyful’. We then categorized stimuli rated 3 in the aesthetic rating and 3 in the emotional rating into ‘joyful beauty’, those rated 3 and 1 into ‘sorrowful beauty’, those rated 2 and 2 into ‘neutral’ and those rated 1 and 2 as ‘ugly’.

### ROI Analysis

Since, we hypothesised the involvement of the mOFC during the experience of both sorrowful and joyful beauty, we constructed a region‐of‐interest (ROI) in the mOFC centered on the coordinates at which activity was found in previous studies to correlate with the experience of beauty derived from visual sources, field A1 (–6 41 −11) [Ishizu and Zeki, 2011], to learn whether activity there also correlates with the experience of two differently valenced emotions which have beauty as a common denominator. We created a ROI mask with 10‐mm sphere to extract average contrast estimates from the mOFC ROI across subjects. We then compared activity within this region between sorrowful beauty > ugliness, as well as joyful beauty > ugliness, against zero, to learn whether mOFC was active with both types of beauty experience. We also compared these two contrasts directly to learn whether the strength of activity differed significantly during the experience of the two emotionally distinct kinds of beauty.

### Whole Brain Analysis

We also conducted whole brain categorical analyses to chart brain activations unique to joyful beauty and sorrowful beauty separately, using the contrasts of sorrowful beauty > ugliness and joyful beauty > ugliness, respectively.

To characterize common brain responses involved in the above two contrasts, we conducted a conjunction analysis performed by a test for independently significant effects as in a logical AND ([sorrowful beauty > ugliness] ∩ [joyful beauty > ugliness]) based on the minimum statistic [Nichols et al., 2005]. Since we had an *a priori* assumption of the involvement of the mOFC in the experience of beauty [e.g., Ishizu and Zeki, 2011, 2013], we used a small volume correction (SVC) on the mOFC with a 16 mm sphere centred on coordinates (–6 41 −11), taken from Ishizu and Zeki [2011].

### Functional Connectivity Analysis

In addition to the regional activity analyses, we also studied the functional connectivity between mOFC and other brain regions to determine the contribution that the latter may make to modulating mOFC activity as a function of the aesthetic and emotional valence of the stimulus, either through input to it or output from it. For this, we performed a psychophysiological interaction (PPI) [Friston et al., 1997]; this tests which regions show activation patterns that co‐vary with mOFC activity, when stimuli are rated as joyfully (or sorrowfully) beautiful or ugly. We assessed changes in functional connectivity between the seed region in the mOFC and other brain regions in two contrasts: joyful beauty > ugliness and sorrowful beauty > ugliness; the analysis for each was performed separately. The PPI employed a design matrix consisting of three regressors representing (1) the extracted time‐series of neural activity within an 8‐mm spherical region centred on the mOFC (‘physiological variable’); (2) a second regressor representing the psychological context of interest, that is, joyful beauty > ugliness and sorrowful beauty > ugliness (‘psychological variables’); (3) a third regressor representing the interaction of the two previous variables (i.e., the interaction between the psychological and physiological variables ‐ ‘PPI term’). Head‐movement parameters were also included in the model as regressors of no interest. This enabled us to identify areas in which the correlation in BOLD activity with the mOFC seed region increases during trials in which a participant experienced sorrowful (or joyful) beauty relative to those during which they experienced ugliness. The PPI was carried out separately for each subject and entered into random‐effects group level analysis. To obtain the data for the physiological variable, we de‐convolved the time‐series of neural activity from the 8‐mm ROI within the mOFC, centred on the coordinates of subject‐specific activations in the region. Thus, to define the ROI, we used the contrast (joyful beauty + sorrowful beauty) > ugliness to locate the closest local maximum to the coordinates [0 48 −16] which we obtained in the group‐level analysis. The results of this analysis are displayed in Table 1 and Figure 6. It should be noted that, although we selected the mOFC as the seed region, the PPI cannot determine directly the directionality of the connectivity or the interaction between the seed region and other brain regions; our discussion is, therefore, limited to interactions between the mOFC and functionally connected regions without specifying their polarity.

**Table 1:** Location, MNI coordinates, cluster size and values for the activations produced by the categorical contrasts of sorrowful beauty > ugliness, joyful beauty > ugliness, sorrowful beauty > joyful beauty, joyful beauty > sorrowful beauty, and by the conjunction analysis sorrowful beauty > ugliness ∩ joyful beauty > ugliness

Cluster p(FWE‐cor)	p(FDR‐cor)	equivk	p(unc)	peak p(FWE‐cor)	p(FDR‐cor)	*T*	equivZ	p(unc)	X	Y	Z (mm)	
Categorical contrasts:												
Sorrowful beauty > Ugliness												
0.004	0.009	137	0.001	0.027	0.249	6.971	4.853	0.000	33	47	7 R	MFG
				0.092	0.255	6.182	4.523	0.000	42	50	10	IFG
				0.132	0.255	5.949	4.417	0.000	45	44	22	dIPFC
0.001	0.004	178	0.000	0.058	0.255	6.475	4.650	0.000	−6	−10	25 L	Caudate
				0.558	0.521	4.894	3.889	0.000	−6	−34	31	PCC
				0.563	0.521	4.885	3.884	0.000	9	−13	25	
0.010	0.016	110	0.001	0.177	0.256	5.755	4.326	0.000	36	−52	−32 R	Cereberum (lobule VI)
				0.208	0.268	5.647	4.274	0.000	27	−46	−26	
				0.333	0.373	5.315	4.110	0.000	21	−40	−26	
0.019	0.023	94	0.003	0.719	0.555	4.628	3.741	0.000	6	38	28 R	dACC
				0.861	0.605	4.360	3.585	0.000	0	38	16	
Joyful beauty > Ugliness												
0.000	0.000	897	0.000	0.000	0.010	9.551	5.715	0.000	−39	−34	−5 L	Hippocampus
				0.001	0.015	8.734	5.472	0.000	15	−19	28	Caudate
				0.012	0.064	7.463	5.041	0.000	21	−37	7	Hippocampus
0.000	0.000	739	0.000	0.021	0.064	7.168	4.930	0.000	3	35	13 R	pgACC
				0.042	0.084	6.716	4.750	0.000	−3	20	22	
				0.137	0.118	5.962	4.423	0.000	3	26	22	
0.072	0.122	60	0.010	0.021	0.064	7.167	4.929	0.000	−36	−64	−38 L	Cerebellum(Lobule VII)
				0.054	0.084	6.561	4.686	0.000	−27	−58	−41	
0.019	0.041	89	0.003	0.234	0.155	5.606	4.255	0.000	30	−58	−38 R	Cerebellum(Lobule VII
				0.374	0.229	5.267	4.086	0.000	39	−61	−38	
				0.411	0.229	5.194	4.048	0.000	27	−49	−29	
0.000	1.000	136	0.000	0.003	0.214	5.928	4.407	0.000	−6	47	−17 L	mOFC
				0.004	0.214	5.726	4.313	0.000	−3	41	−20	
				0.013	0.265	5.004	3.948	0.000	−3	47	−8	
				0.058	1.000	4.094	3.424	0.000	−3	44	4	
0.046	1.000	7	0.336	0.030	0.335	4.506	3.671	0.000	−9	32	1 L	rACC
Sorrowful beauty > Joyful beauty												
0.001	0.001	229	0.000	0.068	0.256	4.783	4.458	0.000	−30	−64	52 L	SPL
				0.155	0.256	4.505	4.228	0.000	−39	−55	49	IPL
				0.585	0.416	3.940	3.746	0.000	−12	−61	52	Precuneus
0.018	0.016	112	0.003	0.182	0.256	4.449	4.181	0.000	30	23	28 R	MFG
				0.425	0.341	4.105	3.889	0.000	45	32	31	
				0.955	0.739	3.450	3.314	0.000	45	23	40	
0.004	0.005	163	0.001	0.195	0.256	4.423	4.159	0.000	−48	32	25 L	MFG
				0.430	0.341	4.100	3.884	0.000	−27	23	28	
				0.477	0.341	4.049	3.841	0.000	−36	29	28	
Joyful beauty > Sorrowful beauty												
0.032	0.117	96	0.006	0.148	0.480	4.522	4.242	0.000	51	−28	25 R	TPJ/SMG
Conjunction([joyful beauty > ugliness] ∩ [sad beauty> ugliness])												
0.436[SVC, −6 41 −1]	0.547L (mOFC)]	28	0.104	0.028	0.155	5.056	4.679	0.000	−36	−64	−35 L	Cerebellum VII
0.023	0.211	16	0.211	0.017	0.335	3.933	3.740	0.000	−6	35	−17 L	mOFC
				0.026	0.335	3.772	3.600	0.000	−12	38	−14	
PPIs:												
mOFC_Sorrowful beauty > Ugliness												
0.000	0.000	1,466	0.000	0.000	0.004	10.330	5.926	0.000	39	−82	4 R	Middle occipital gyrus
				0.002	0.030	8.310	5.336	0.000	−3	−82	1	Lingual gyrus
				0.004	0.030	8.092	5.263	0.000	3	−76	1	
0.000	0.001	282	0.000	0.547	0.547	4.754	3.812	0.000	−6	−4	61 L	SMA (encroaching to MCC)
				0.580	0.550	4.699	3.781	0.000	6	11	67	
				0.722	0.658	4.464	3.646	0.000	−9	5	46	
[SVC, 33 47 1	' (dIPFC)]											
0.040	0.393	8	0.393	0.024	0.236	4.450	3.640	0.000	33	53	16 R	dIPFC/MFG
mOFC_Joyful beauty > Ugliness												
0.000	0.000	886	0.000	0.004	0.037	8.484	5.238	0.000	51	−61	−2 R	MTG
				0.076	0.125	6.610	4.591	0.000	9	−88	1	
				0.134	0.125	6.199	4.423	0.000	15	−88	−5	
0.014	0.009	110	0.002	0.068	0.125	6.690	4.622	0.000	−9	62	10 L	rMPFC
				0.866	0.387	4.459	3.579	0.000	6	59	19	
				0.985	0.685	3.961	3.288	0.001	−12	50	16	
0.000	0.000	612	0.000	0.133	0.125	6.202	4.425	0.000	−42	−73	−11 L	Occipital gyrus
				0.182	0.125	5.974	4.327	0.000	−27	−79	−17	
				0.333	0.131	5.509	4.117	0.000	−36	−67	−8	
| 0.013	0.009	112	0.002	0.188	0.125	5.950	4.317	0.000	−39	−13	52 L	Precentral gyrus
				0.192	0.125	5.934	4.310	0.000	−48	−19	49	
				0.955	0.548	4.168	3.412	0.000	−30	−22	64	

Also shown are areas exhibiting greater functional connectivity with the mOFC in psychophysiological interactions (PPIs), in the contrasts sorrowful beauty > ugliness and joyful beauty > ugliness.

We report cluster level activations significant at *P* < 0.05 family‐wise error (FWE) corrected, although some of these (indicated in the table) were also significant at the peak level at *P* < 0.05 FWE corrected. The coordinates of all activations are reported in MNI space.

## RESULTS

### Behavioral Results

**Figure 2:**
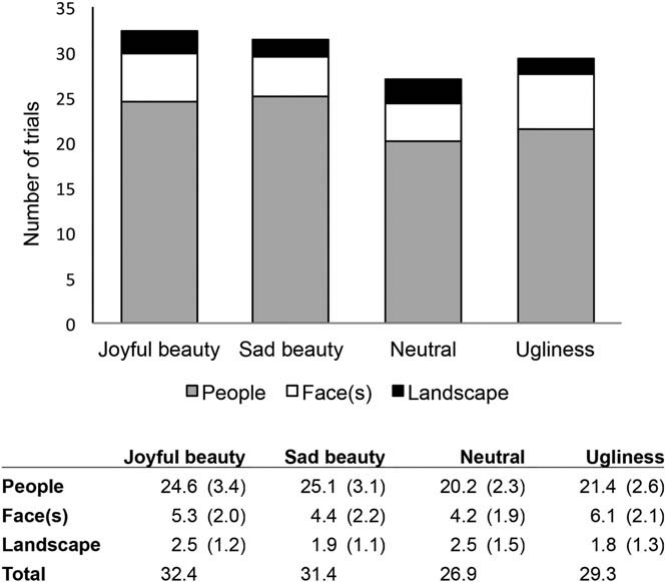
Behavioral data collected in the fMRI experiment, showing averaged number of trials with each kind of stimulus (people, faces, and landscape without a human figure), with standard deviations for each of the four experimental conditions across 20 subjects.

Ideally, the status of the ratings given to the 120 stimuli viewed in the scanner should be the same as that given during the preliminary viewing test, that is, each condition (e.g., beautiful *and* sorrowful) should appear 30 times. In reality, such an ideal situation was not reached. Based on the aesthetic ratings given during the scanning sessions and the postscanning emotional ratings, we obtained the following ratings over 20 participants: 32.4 for ‘joyful beauty’ (3–3), 31.4 for ‘sorrowful beauty’ (3–1), 26.9 for ‘neutral’ (2–2), and 29.3 for ‘ugliness’ (2–1) (Fig. 2), on average. These slight variations in the ratings during the preliminary tests and during the scanning functional magnetic resonance imaging (fMRI) session are not of great concern since there was a reasonably distributed number of trials in each condition. The stimuli consisted of pictures of a face(s), people including faces, and landscapes without human figures. Figure 2 shows details of the number of images in terms of stimulus contents. It is known that viewing different types of visual stimuli, for example, faces versus scenes, results in activation of different brain areas, reflecting the functional specialization of the visual brain [Kawabata and Zeki, 2004; Zeki et al., 1991]. All four experimental conditions in this study, however, had similar proportions of images belonging to the different categories, that is, human figures (including faces and people) and landscapes (see Fig. 2). The most prevalent stimulus images we had for each subject contained human figures, that is, people or faces (92.7% on average (90.7–93.9%) across the experimental conditions), whereas, very few consisted of scenes without a human figure (7.3% on average (6.1–9.3%)). We conducted a 2‐way analysis of variance with 2 stimulus contents (human figure, landscape) and 4 response conditions (joyful beauty, sad beauty, neutral, ugliness). There was no significant difference in response conditions and interactions, while a main effect of stimulus contents alone was observed (df = 19, *F* = 1,631, *P* < 0.001). Therefore, the brain responses found by contrasting the conditions we report here cannot be explained by differences in stimulus contents.

### Neuroimaging Results

#### mOFC

**Figure 3:**
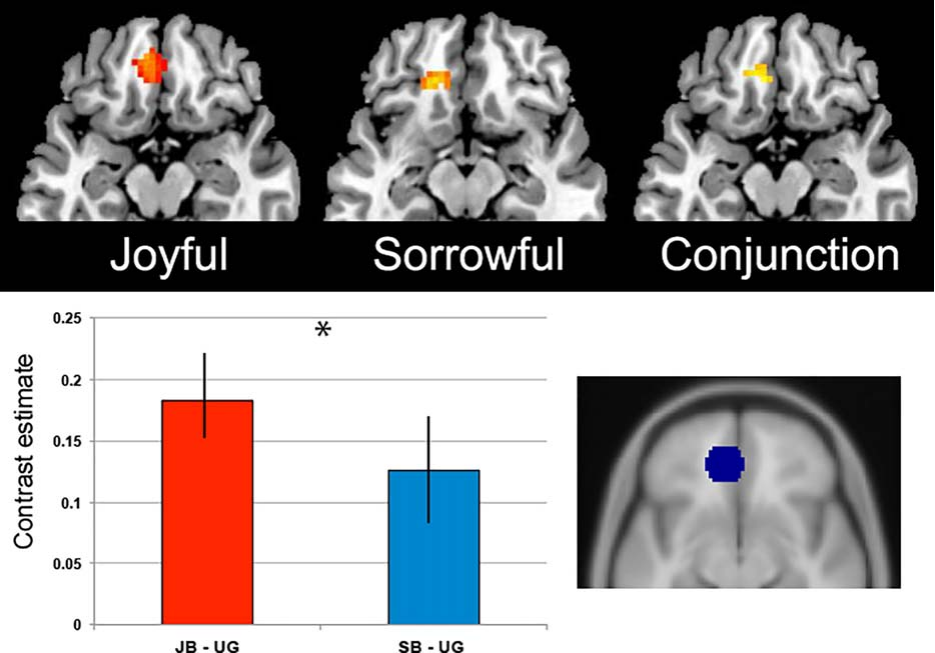
The upper panel shows the activation within the medial orbitofrontal cortex correlating with the experience of joyful and sorrowful beauty. Statistical parametric maps rendered onto canonical anatomical sections showing the *t*‐statistic for (left) joyful beauty > ugliness, (middle) sorrowful beauty > ugliness, and (right) the results of a conjunction analysis for joyful beauty > ugliness ∩ sorrowful beauty > ugliness. Random effects analysis with 20 subjects. Display threshold *P* < 0.001 (uncorrected). (Lower right) Region of interest in the mOFC. (Lower left) Averaged contrast estimates for the contrasts joyful beauty > ugliness (JB > UG) and sorrowful beauty > ugliness (SB > UG) within the defined ROI (–6 41 −11), over 20 subjects. Joyful beauty caused a higher BOLD signal than sorrowful beauty. * *P* < 0.05. Error bars are standard error (SE). [Color figure can be viewed at http://wileyonlinelibrary.com]

To learn whether there was a difference in the strength of activity within the mOFC between the experience of sorrowful and joyful beauty, we extracted, for each subject, the contrast estimates within the defined ROI in the mOFC and compared across conditions. First, one sample *t*‐tests revealed that the extracted averaged contrasts estimates for ‘sorrowful beauty’ and ‘joyful beauty’, both against ‘ugliness’, were different from zero (sorrowful beauty, *t* = 24.3, df = 19, *P* < 0.001; joyful beauty, *t* = 39.7, df = 19, *P* < 0.001), showing that, as we expected, the mOFC is engaged during the experience of both joyful beauty and sorrowful beauty. This area has been reported to be active in previous studies on aesthetic experiences using a variety of stimuli [Ishizu and Zeki, 2011; Kawabata and Zeki, 2004; O'Doherty et al., 2003; Tsukiura and Cabeza, 2011; Zeki et al., 2014]. A direct comparison between the two conditions showed, however, that the averaged contrast estimates for joyful beauty gave higher values than that for sorrowful beauty (paired *t*‐test, *t* = 10.4, df = 38, *P* < 0.001) (Fig. 3).

**Figure 4:**
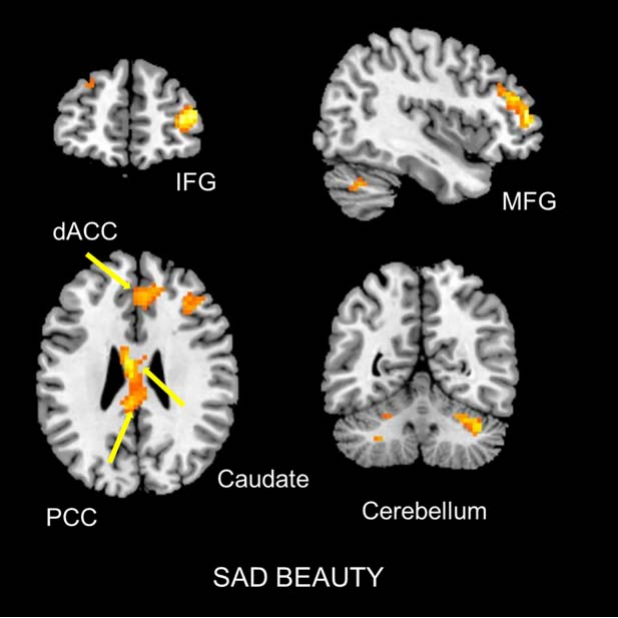
Sites active during the experience of sorrowful beauty. Statistical parametric maps rendered onto canonical anatomical sections showing the *t*‐statistic for the contrast sorrowful beauty > ugliness. Random effects analysis with 20 subjects. Display threshold *P* < 0.001 (uncorrected). Abbreviations: IFG, frontal gyrus; MFG, middle frontal gyrus; dACC, dorsal anterior cingulate cortex; PCC, posterior cingulate cortex. [Color figure can be viewed at http://wileyonlinelibrary.com]

**Figure 5:**
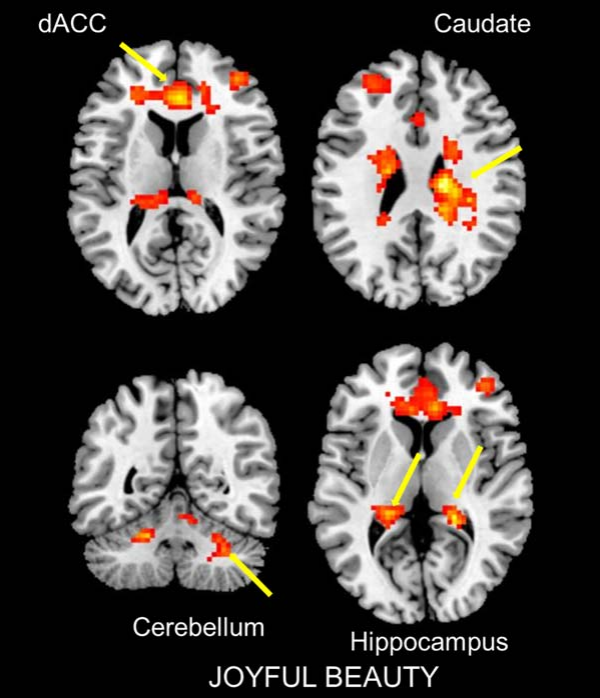
Sites active during the experience of joyful beauty. Statistical parametric maps rendered onto canonical anatomical sections showing the *t*‐statistic for the contrast joyful beauty > ugliness. Random effects analysis with 20 subjects. Display threshold *P* < 0.001 (uncorrected). Abbreviation: dACC, dorsal anterior cingulate cortex. [Color figure can be viewed at http://wileyonlinelibrary.com]

In short, the ROI results suggest that the mOFC is commonly activated with the experience of beauty but that the strength of the activation may be modulated by the valenced emotions.

#### Whole brain contrasts

Our main aim was to learn whether the brain regions previously reported as active during the experience of beauty, and in particular the mOFC, were differentially engaged during the experience of sorrowful and joyful beauty. But we were also interested in learning whether, besides the mOFC, significantly different brain regions were involved when the experience was that of sorrowful as opposed to joyful beauty, especially given that the two arouse different empathetic feelings.

To chart brain activations that correlate with the experience of sorrowful and joyful beauty, we performed separate categorical contrasts of (1) sorrowful beauty > ugliness, and of joyful beauty > ugliness; we also contrasted the activity produced by (2) joyful beauty versus sorrowful beauty, to directly compare brain regions that are uniquely active during each kind of aesthetic experience. We then used a conjunction analysis [Nichols, et al., 2005] to characterize brain activations common to both sorrowful and joyful beauty using the contrast [sorrowful beauty > ugliness] ∩ [joyful beauty > ugliness]. All activations are listed in Table 1.

##### Sorrowful beauty versus ugliness

The contrast sorrowful beauty > ugliness resulted in activity in lateral frontal lobe including the middle frontal gyrus (MFG); this band of activity extended to the inferior frontal gyrus (IFG) and the dorso‐lateral prefrontal cortex (dlPFC). The posterior cingulate cortex (PCC), encroaching upon the caudate (head and body), was also active. In addition, there was activity in parts of the cerebellum (lobule VI) and dorsal ACC (see Figs. 4, 7, and 8).

In short, some cortical regions which previous studies had found to be active during sorrowful experiences were also active in the contrast of sorrowful beauty versus ugliness.

##### Joyful beauty versus ugliness

The contrast joyful beauty *>* ugliness led to activation in the right mOFC, in a region adjoining rostro‐ventral anterior cingulate cortex (ACC); this entire zone has been reported to be active in previous studies of aesthetic experiences [e.g., Ishizu and Zeki, 2011; Tsukiura and Cabeza, 2011; Zeki et al., 2014]. The body of the right caudate nucleus and pregenual ACC (pgACC), which have been found to be active in the experience of visual beauty and aesthetic and evaluative judgments [Cunningham et al., 2004; Ishizu and Zeki, 2011; Jacobsen et al., 2006; Vartanian and Goel, 2004], were also active. In addition, there were activations in bilateral posterior hippocampus and parts of the cerebellum (lobule VII crus I and II) (see Figs. 5, 7, and 8).

In short, in addition to the activation within the mOFC, we observed a similar pattern of cortical and subcortical activations in the contrast of joyful beauty versus ugliness to that reported in previous studies of visual beauty.

##### Areas uniquely active for each kind of beauty

The contrast sorrowful > joyful beauty produced activation in: the left inferior parietal lobe (IPL) encroaching on the precuneus, a region which has often been observed in studies of emotional/social pain (e.g., Lamm et al., 2011); it also produced activity in bilateral MFG, parts of which have been found to be involved during emotional states [Acevedo et al., 2014; Sabatinelli et al., 2011].

**Figure 6:**
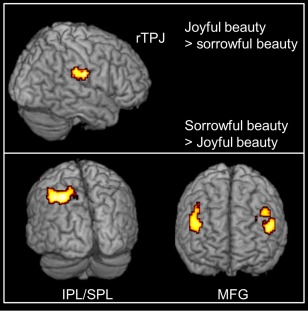
Sites revealed to be active in the contrast sorrowful versus joyful beauty. Statistical parametric maps rendered onto canonical anatomical sections showing the *t*‐statistic for (upper) the contrasts joyful beauty > sorrowful beauty and (lower) sorrowful beauty > joyful beauty. Random effects analysis with 20 subjects. Display threshold *P* < 0.001 (uncorrected). Abbreviations: rTPJ, right temporoparietal junction; IPL, inferior parietal lobe; SPL, superior parietal lobe; MFG, middle frontal gyrus. [Color figure can be viewed at http://wileyonlinelibrary.com]

The reverse contrast, of joyful > sorrowful beauty led to activation in the right temporoparietal junction (TPJ), including the supramarginal gyrus (SMG), a region thought to be involved in controlling empathy toward others [e.g., Silani et al., 2013] (Fig. 6).

In summary, the experience of joyful and sorrowful beauty had, as correlates, distinct patterns of cortical activity. The former included the TPJ and the SMG alone while the latter included the left parietal lobe, the precuneus, and bilateral MFG.

##### Areas commonly active during the experience of both types of beauty

The conjunction analysis ([sorrowful beauty > ugliness] ∩ [joyful beauty > ugliness]) showed common activation in the mOFC with the application of SVC, and left cerebellum (lobule VII), whereas the ACC, which was active in both conditions, did not survive this threshold in the conjunction analysis (Fig. 3).

### Functional Connectivity

The conjunction analysis revealed that the mOFC is engaged during the experience of both sorrowful and joyful beauty. We undertook a PPI analysis to learn more about the possible differential neural engagements during the experience of the two types of beauty, by examining the functional connectivity between the mOFC and other brain regions. Because the stimuli experienced as being sorrowful contained, for the most part, pitiful and empathetic scenes (such as pictures of funerals, an abandoned child, a crying man, separations, etc.), we expected that, in this condition, the mOFC will show greater functional connectivity with regions related to the experience of sad (negative) emotional empathy and the interpretation of others’ intentions, especially under sad or painful situations (‘negative empathy’ as opposed to ‘positive empathy’ [Morelli et al., 2015]); these regions are the middle cingulate cortex (MCC), the supplementary motor area (SMA) [Fan et al., 2011], and the dlPFC [Lieberman, 2007 for a review; Weissman et al., 2008]. Using sorrowful beauty and ugliness as the psychological parameters, we tested whether the physiological coupling between mOFC, as the seed region, and other regions besides the ones mentioned above, would change between the experience of sorrowful beauty and ugliness. The results showed that there was indeed increased functional connectivity between the mOFC and SMA, encroaching onto MCC, and several other regions (see Fig. 9 and Table 1). With the application of SVC using the coordinates based on a previous PPI study in the bilateral dlPFC [Kirk et al., 2011], dlPFC also showed a significant increased connectivity with the mOFC during the experience of sorrowful beauty. We then compared the averaged *β*‐value in the SMA/MCC during the experience of sorrowful and joyful beauty and found a stronger connectivity between the SMA/MCC and the mOFC in sorrowful beauty relative to joyful beauty (*t*(19) = 4.03, *P* < 0.01).

In contrast, the experience of joyful beauty produced increased functional connectivity between the mOFC and the anterior rostral medial prefrontal cortex (anterior rMPFC) and middle temporal gyrus (MTG), among several other regions (see Table 1). The averaged *β*‐value in the rMPFC showed stronger connectivity in joyful beauty than sorrowful beauty (*t*(19) = 5.31, *P* < 0.01). All other PPI results are listed in Table 1.

## DISCUSSION

The experience of beauty may, in general terms, be regarded as a positive, rewarding, and pleasurable one. It is perhaps not surprising, therefore, that regardless of source, it correlates with activity in field A1 of mOFC [Ishizu and Zeki, 2011], a region of the emotional brain which has been generally associated with pleasure and reward (e.g., O'Doherty et al., 2001); activity in it has been shown to correlate parametrically with the declared intensity of the experience of beauty derived from a variety of stimuli, such as faces, colors, motion, paintings, music, architectures, moral judgments, and mathematical equations [Ikeda et al., 2015; Ishizu and Zeki, 2011; Kawabata and Zeki, 2004; Kuhn and Gallinat, 2012; O'Doherty et al., 2003; Zeki and Stutters, 2012; Tsukiura and Cabeza, 2011; Vartanian et al., 2013 for a meta‐analysis; Zeki et al., 2014] though apparently not from the performing (dance) arts [e.g., Calvo‐Merino, et al., 2008; Cross et al., 2011]. Moreover, a recent study has reported an increase in aesthetic ratings of visual stimuli [Nakamura and Kawabata, 2015] following the application of anodal transcranial direct current stimulation to the mOFC, presumably because of enhanced neural activity within it.

In the work reported here, we wanted to go a step beyond and enquire into whether the experience of beauty linked to different and indeed opposite emotional states would also correlate with activity in A1 of mOFC. Sorrowful beauty is commonly associated with negative empathy while joyful beauty is linked to positive empathy. The two experiences studied here are, thus, associated with opposite empathetic sources but share a common denominator, that of beauty. Given that field A1 of mOFC correlates with the experience of beauty regardless of source, it was natural to hypothesize that the experience of beauty linked to emotional states of opposite valence would also correlate with activity in the same area, which is indeed what we found. But the intensity of activity in A1 of mOFC (defined as the ROI) was greater during the experience of beauty derived from joy than that derived from sorrow. This made it interesting to enquire into the strength of connectivity between the mOFC and areas of the brain that have been associated with experience of two opposite states of empathy.

### Negative and Positive Empathy Reflected in the Pattern of Brain Activity

Empathy has been studied in fair detail recently but most do not refer explicitly to a distinction between negative and positive empathy. That the two are separate is, of course, a common human experience; it is reflected here in the distinct patterns of activation that correlate with the experience of sorrowful and joyful beauty, besides the common correlate in activity of A1 of mOFC. This distinction can be discerned in (a) the general pattern of cortical activity, (b) in activity within the anterior cingulate cortex, and (c) in the cerebellum.

a. *General cortical activation patterns*. Contrasting the pattern produced by the experience of sorrowful beauty with that produced by the experience of ugliness resulted in an extensive pattern of activity that includes, in addition to the reward‐related regions, bilateral MFG, extending to IFG, right dlPFC and PCC; all three areas are known to be active when empathizing with others, especially in negative emotional conditions. The IFG, in particular, has been reported to be active during listening to minor chords (rated as sad and indicative of sorrow) compared to major ones (which were rated as ‘happy’), even though both were rated as aesthetically beautiful by the subjects [Suzuki et al., 2008]. This region has also been reported to be active when viewing pictures of humans suffering from harm and threat [Nummenmaa et al., 2008], while the MFG has been reported to be active during the viewing of sad faces [Acevedo et al., 2014; Sabatinelli et al., 2011]. The dlPFC has been linked to handling complex social situations [Lieberman, 2007 for a review; Weissman et al., 2008], control of emotional states [Goldin et al., 2008; Keightley et al., 2003], inferring others’ intentions, and theory of mind [Guroglu et al., 2011]. The PCC, encroaching upon the caudate (head and body), was also active; this region has been implicated in a range of functions including the experience of high valence emotional stimuli [Maddock et al., 2003], theory of mind [Fletcher et al., 1995; Greene et al., 2001], and sad autobiographical recall [Farrow et al., 2001; Maddock, 1999]. Activity in the IPL, also observed with the experience of sorrowful beauty when contrasted with joyful beauty, has been associated with emotional or social pain and, together with the IFG, has been considered as constituting an ‘emotion contagion network’ underlying our ability to empathize emotionally [Shamay‐Tsoory, 2011], though negatively.

**Figure 7:**
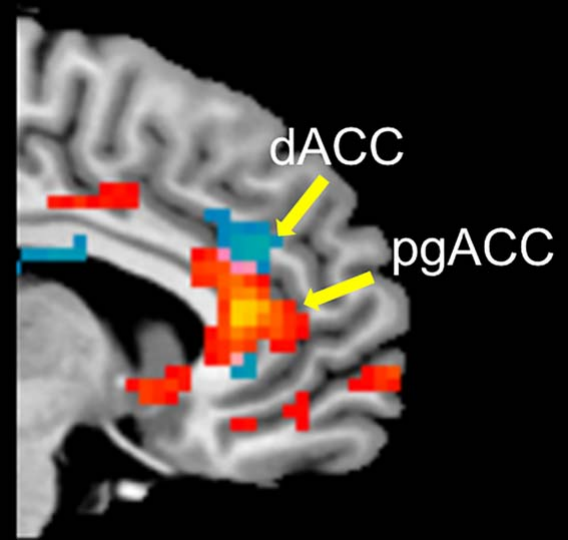
Sites active within the ACC during the experience of sorrowful and joyful beauty. Statistical parametric maps rendered onto canonical anatomical sections showing the *t*‐statistic for (red) the contrasts joyful beauty > ugliness and (blue) sorrowful beauty > ugliness. Random effects analysis with 20 subjects. Display threshold *P* < 0.001 (uncorrected). Abbreviations: dACC, dorsal anterior cingulate cortex; pgACC, perigenual anterior cingulate cortex. [Color figure can be viewed at http://wileyonlinelibrary.com]

**Figure 8:**
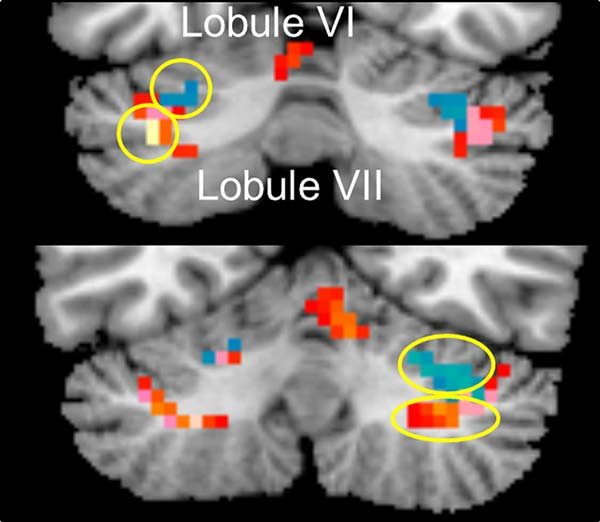
Sites active within the cerebellum during the experience of sorrowful and joyful beauty. Statistical parametric maps rendered onto canonical anatomical sections showing the *t*‐statistic for (red) the contrasts joyful beauty > ugliness and (blue) sorrowful beauty > ugliness. Random effects analysis with 20 subjects. Display threshold *P* < 0.001 (uncorrected). [Color figure can be viewed at http://wileyonlinelibrary.com]

By contrast, the experience of joyful beauty correlated with activity in the right TPJ and in the SMG, both of which have been considered to be involved in controlling empathy toward others, by overriding an emphasis on the self (ego‐centricity) [e.g., Silani et al., 2013]. Activity in the TPJ, which has been considered to play an important role in interpersonal emotional and cognitive interactions [Saxe, 2006, for a review], has been reported to increase when subjects view happy faces compared to angry or disappointed ones [Lelieveld et al., 2013] and both TPJ and SMG have been demonstrated to be part of a larger cortical zone, which includes the right parietal area, that is active when adopting other peoples’ emotional states [e.g., Ruby and Decety, 2004].

b. *Anterior cingulate cortex*: Activation within the ACC found during the experience of sorrowful and joyful beauty can be separated into dorsal (dACC) for the former and the pregenual subdivision (pgACC) for the latter (see Fig. 7). It has been suggested that the dACC is active during the experience of emotionally distressing conditions such as physical and social pain (acknowledging others’ pain) [Eisenberger and Lieberman, 2004; Lamm et al., 2011 for a review], whereas the pgACC activity (including activity in the adjacent subgenual ACC and mOFC/ventromedial prefrontal cortex (vmPFC)) correlates with the experience of positive emotions [Etkin et al., 2011 for a review]. A previous study reports activity in this region when viewing aesthetically pleasing stimuli [e.g., Vartanian et al., 2013]. ACC's diverse cognitive and emotional functions make it difficult to define each subdivision's involvement in a precise function. But previous studies suggest that the ventral and sub/pregenual areas are involved in processing of emotion, especially positive emotion while the dorsal subdivision, by contrast, is strongly associated with negative emotional states as well as cognitive components. This separation, again, reflects anatomically the involvement of separate regions in positive and negative components of aesthetic experiences.

c. *Cerebellum*: The dichotomy in neural activity that correlates with the two contrasting experiences is also reflected in cerebellar activity. We found that the experience of sorrowful and joyful beauty engaged different parts of the cerebellum, lobule VI for sorrowful beauty and lobule VII for joyful beauty (see Fig. 8). Although there is no current consensus regarding the pattern of cerebellar activity during aesthetic experiences, several past studies have reported activity in cerebellum during aesthetic experiences, including ones derived from visual and literary beauty as well as during the judgment of beauty [Bohrn et al., 2013; Ishizu and Zeki, 2013; Vartanian and Goel, 2004]. It is noteworthy that sorrowful beauty engaged lobule VI, which past studies have shown to be more responsive to negatively charged stimuli such as sadness, fear, and anger than to positive ones [Baumann and Mattingley, 2012; Park et al., 2010] and to perspective taking to others’ pain [Lamm et al., 2007], which can be regarded as negative empathy in the context of the current study. Although the cerebellum is, in general, more active during what may be regarded as negative emotions [Stoodley, 2012], crus II (lobule VII) has been reported to be more strongly engaged when viewing pictures representing happiness than disgust [Schienle and Scharmuller, 2013] and is uniquely active with joyful beauty in the current experiment.

**Figure 9:**
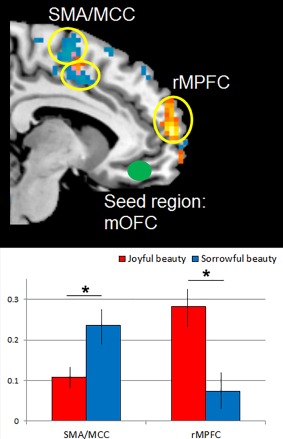
The upper panel shows the functional connectivity observed in this study, with the mOFC as the seed region. Areas in orange (anterior rMPFC) showed greater functional connectivity with the mOFC during the experience of joyful beauty, whereas areas shown in blue (SMA/MCC) showed greater connectivity during the experience of sorrowful beauty. Display threshold *P* < 0.001 (uncorrected). Lower panel (left) shows the averaged beta‐estimates measuring the correlation between BOLD activity in the SMA/MCC and the mOFC. Lower panel (right) shows the averaged beta‐estimates measuring the correlation between BOLD activity in the anterior rMPFC and the mOFC. Blue bars denote the averaged beta‐estimates with sorrowful beauty and red bars denote those with joyful beauty. Error bars are standard error (SE). Abbreviations: SMA, supplementary motor area; MCC, middle cingulate cortex; rMPFC, rostral medial prefrontal cortex; mOFC, medial orbito‐frontal cortex. [Color figure can be viewed at http://wileyonlinelibrary.com]

Patient studies have suggested that lesions in lobule VI and VII can lead to cerebellar cognitive affective syndrome, when patients suffer from various emotional and behavioral deficits, including flattening of emotions or impulsive behaviors [Schmahmann, 2004; Schmahmann et al., 2007]. It has been suggested that those deficits in emotion, possibly produced by disrupting the cerebellar‐limbic connection [Stoodley and Schmahmann, 2009], can affect the ability to communicate and empathize with the perspectives of others [Oberman and Ramachandran, 2007].

Hence, both cortical and cerebellar activations reflect, in a sense, the common human experience which can separate sorrowful from joyful beauty. Even though this is a distinction that is not commonly made or emphasized in philosophies of aesthetics, it is, nevertheless, one that correlates with distinct patterns of activation during the experience of the two different kinds of beauty.

#### Functional connectivity

Given this dichotomy, we naturally expected that some of the areas that were active above would show a positive, state‐dependent positive connectivity with mOFC. It is known that activity in the mOFC (and vmPFC) can be modulated by signals from other brain regions [Harvey et al., 2010]. The areas showing a greater functional connectivity with the mOFC during the experience of sorrowful beauty were the SMA, MCC, and dlPFC, regions related to the experience of sad (negative) emotional empathy and the interpretation of others’ intentions, especially under sad or painful situations [Lieberman, 2007 for a review; Fan et al., 2011; Weissman et al., 2008]. One study using diffusion‐weighted and functional MRI showed a direct connection between the SMA and OFC area [Johansen‐Berg et al., 2004]. It is known that aesthetic judgments under the influence of monetary value and sponsorship to artworks lead to increased functional connectivity between the mOFC and the dlPFC, activity in the former being influenced by that of the latter [Harvey et al., 2010; Kirk et al., 2011]. Hare et al., [2009, 2010] reported that the dlPFC modulated value signals encoded in the vmPFC/mOFC when subjects were given information about the health status of a food item and conducted self‐controlling dietary choice.

Joyful beauty, in contrast, did not exhibit a functional connectivity with the regions relating to negative empathy, but showed a greater connectivity with anterior part of the rMPFC, a region thought to be involved in mentalizing other people's psychological perspective [e.g., Amodio and Frith, 2006 for a review; Skerry and Saxe, 2015]. It is not clear whether different patterns of activity in the rMPFC correlate with the experience of positive and negative emotion in mentalizing but some studies suggest that it responds more to positive and aesthetically pleasing stimuli [Kreplin and Fairclough, 2013; Vessel et al., 2012]. An enhanced functional connection between mOFC/vmPFC, MPFC, and MTG during the judgment of facial attractiveness has been reported in a previous PPI study [Smith et al., 2014].

It has been suggested that the mOFC encodes the aesthetic value on the basis of a common neural scale regardless of its source [e.g., Ishizu and Zeki, 2011; Pegors et al., 2015; Zeki et al., 2014], leading to an enquiry on how relevant information, such as emotional context, modulates perceived aesthetic value and how such a modulation is represented in neural terms, besides activity within the mOFC [Pegors et al., 2015]. The findings from recent studies, some mentioned above, have suggested that the mOFC interacts with other brain systems during evaluation of reward values in a ‘context‐dependent’ manner [Levy and Glimcher, 2012; Smith et al., 2014]. We have revealed that there is an increased functional connectivity between the mOFC and anterior rMPFC in joyful beauty and between the mOFC and the SMA/MCC and the dlPFC in sorrowful beauty. This finding suggests that (1) the brain engages two specialised systems, a reward‐related one (the mOFC) and empathy‐related regions; these are dissociable from each other depending upon whether the experience has a positive or negative emotional valance (the SMA/MCC and rMPFC), and (2) the empathy‐related regions may modulate activity within the mOFC through functional connectivity, to enable us to experience the contradictory aesthetic and emotional values.

In summary, therefore, functional connectivity between mOFC and other cortical areas during the experience of beauty is dictated by whether the experienced beauty is joyful or sorrowful.

It is interesting to discuss briefly the neural correlates of pleasure evoked by listening to sad music, which is also regarded as being a positive aesthetic experience with a negative emotional valence [Schubert, 1996]. Such a contradictory experience can be seen in many forms of art, including paintings and films [e.g., Hanich et al., 2014; Leder et al., 2014] but is most notable and relatively well studied in music. Among previous behavioral and neuroimaging studies on sad music [e.g., Kawakami et al., 2013; Suzuki et al., 2008; Taruffi and Koelsch, 2014], a recent one [Sachs et al., 2015] has argued that sad music is found pleasurable when (1) it is perceived as non‐life‐threatening and with, no immediate real life implication; (2) it is aesthetically pleasing; and (3) it has certain psychological benefits, such as mood regulation caused by recollection of personal past events, which leads to activation within hippocampus/parahippocampal gyrus. We did not find hippocampal activity in the sorrowful beauty condition in the current study; instead, we found activation within SMA/MCC, indicating empathy and perspective taking toward other people. This may be due to a difference in the nature of visual and musical perception. With visual stimuli, viewers can immediately empathize with sufferers or wounded people depicted in an image. By contrast, music, having no figurative representation, may make listeners adopt a more ‘self‐referential mode’ and recall personal‐relevant memories. This points to possible interesting future studies, of how the brain reacts to the experience of sadness evoked by different sources.

## CONCLUSION

It is gratifying to us that an inspiration derived from a literary source should have led to work which has given us a little, but not much, more knowledge about the brain mechanisms that are engaged during aesthetic experiences.

## CONFLICT OF INTEREST

The authors declare no conflict of interest.

## Supporting information

Supporting Information
