# Supplementary figures and images for: The experience of beauty derived from sorrow
Source: Hum Brain Mapp. 2017 May 23;38(8):4185–200. doi: 10.1002/hbm.23657 (PMC5518297; doi:10.1002/hbm.23657)

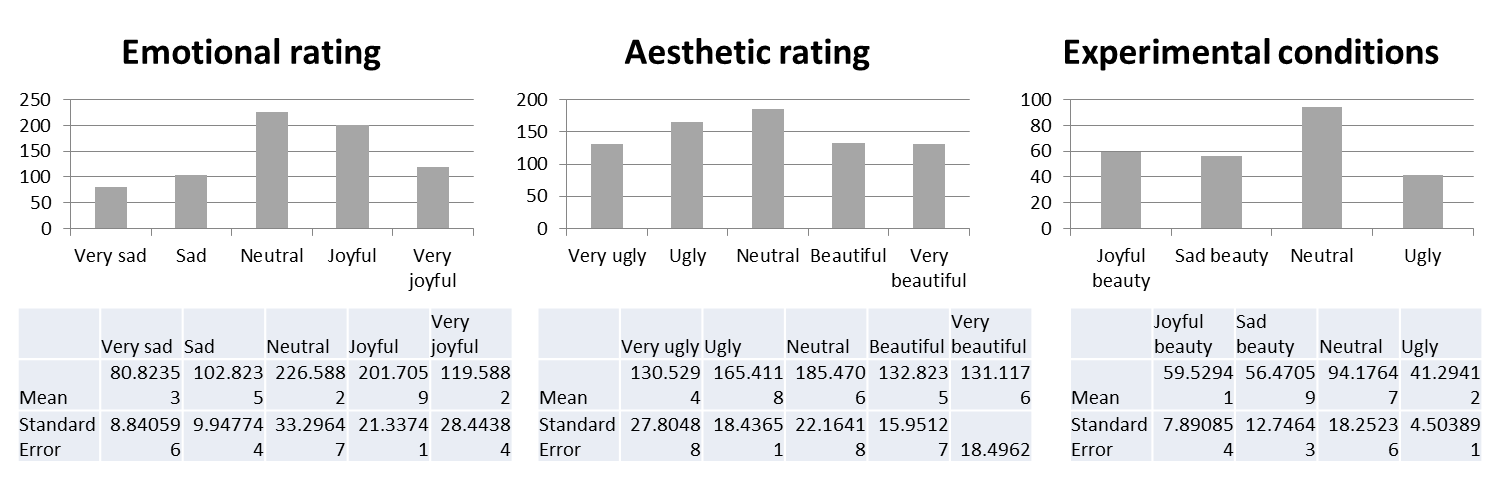

Supplement: Supplementary file 1 — Supporting Information [file HBM-38-4185-s001.tif]
